# Composition of Sugars in Wild and Cultivated Lingonberries (*Vaccinium vitis-idaea* L.)

**DOI:** 10.3390/molecules24234225

**Published:** 2019-11-20

**Authors:** Gabriele Vilkickyte, Raimondas Raudonis, Vida Motiekaityte, Rimanta Vainoriene, Deividas Burdulis, Jonas Viskelis, Lina Raudone

**Affiliations:** 1 Laboratory of Biopharmaceutical Research, Institute of Pharmaceutical Technologies, Lithuanian University of Health Sciences, Sukileliu av. 13, LT-50162 Kaunas, Lithuania; lina.raudone@lsmuni.lt; 2 Department of Pharmacognosy, Lithuanian University of Health Sciences, Sukileliu av. 13, LT-50162 Kaunas, Lithuania; raimondas.raudonis@lsmuni.lt (R.R.); deividas.burdulis@lsmuni.lt (D.B.); 3 Biomedical Sciences Department, Siauliai State College. Ausros av. 40, LT-76241 Siauliai, Lithuania; vmotiek@gmail.com; 4 Paitaiciu str, The Botanical Garden of Siauliai University, 4, LT-77175 Siauliai, Lithuania; rimanta.vainoriene@su.lt; 5 Laboratory of Biochemistry and Technology, Institute of Horticulture, Lithuanian Research Centre for Agriculture and Forestry, Kauno str. 30, LT-54333 Babtai, Kaunas distr., Lithuania; jonas.viskelis@lammc.lt

**Keywords:** *Vaccinium vitis-idaea*, lingonberry, sugars, cultivated berries, wild berries, HPLC–ELSD

## Abstract

Products of lingonberries are widely used in the human diet; they are also promising beauty and health therapeutic candidates in the cosmetic and pharmaceutical industries. It is important to examine the sugar profile of these berries, due to potential deleterious health effects resulting from high sugar consumption. The aim of this study was to determine the composition of sugars in wild clones and cultivars or lower taxa of lingonberries by HPLC–ELSD method of analysis. Acceptable system suitability, linearity, limits of detection and quantification, precision, and accuracy of this analytical method were achieved. The same sugars with moderate amounts of fructose, glucose, and low amounts of sucrose were found in wild and cultivated lingonberries. Cultivar ‘Erntekrone’ and wild lingonberries collected from full sun, dry pine tree forests with lower altitude and latitude of the location, distinguished themselves with exclusive high contents of sugars. The changes in the sugar levels during the growing season were apparent in lingonberries and the highest amounts accumulated at the end of the vegetation. According to our findings, lingonberries seem to be an appropriate source of dietary sugars.

## 1. Introduction

As the modern lifestyle and prevalence of chronic diseases have become a relevant issue of concern, functional food ingredients, nutraceuticals, and food supplements have become a significant area of research. Increasing sugar intake is of concern, because it may result in obesity, metabolic disorders, dental caries, and increased risk of noncommunicable diseases—hypertension, dyslipidemia, cardiovascular diseases, type 2 diabetes, cancer and others, which are responsible for more than half of all deaths worldwide [1,2]. Possible harmful cardiometabolic mechanisms of action of dietary sugars include induction of inflammatory processes, oxidative stress, increasing insulin resistance, and impaired β-cell function [3]. Sugars differ from each other in structure, effects, and applications. According to the structure, sugars can be referred to as: (1) Monosaccharides (glucose, fructose, and galactose), which have five or six carbon atoms, sweet taste and can be called reducing sugars; (2) disaccharides (sucrose, lactose, maltose, trehalose), which make up two or more monosaccharide units and may not have reducing properties; and (3) polyols—sugar alcohols (sorbitol, mannitol, lactitol, xylitol, erythritol, isomalt, maltitol). Sugars may naturally present in food or can be added to food additionally by the manufacturer or consumer. The latter is considered to be more dangerous because of the faster absorption and conversion to fats [4,5,6].

Deleterious health effects may occur if sugars are consumed in large amounts [3]. The same can be said about any macro- or micronutrient. The moderate intake of sugars, which according to WHO should be less than 10% of total energy intake, does not increase the health risk [1]. While excessive amounts of sugars are undoubtedly unhealthy, rational use of sugars can be favorable. Sugars are a prominent constituent of plants, acting as structure matter and molecule signal, regulating growth and enzyme activity [7]. Furthermore, they are the main energy source for the human body and an ubiquitous ingredient of our food, providing a desirable sweet taste [4,8].

It has been suggested that some sugars take part in the anti-adherence activity. Adhesin proteins, fimbriae or pili, expose adhesive lectins on the cell surface, which bind complementary carbohydrates on the tissues of the host, and thus permit *Escherichia coli* bacterial adhesion to the urothelium. Adhesin proteins can be mannose-resistant (p-fimbriae) or mannose-sensitive (type 1 fimbriae). The current hypothesis proposes that proanthocyanidins inhibit the adherence of p-fimbriae, and fructose inhibits adherence of type 1 fimbriae; consequently, uropathogenic bacteria cannot infect mucosal surface and postulate urinary tract infections [9,10]. Hereby, fructose contributes to the disease-preventing properties of the most common bacterial infections, acquired in the community or hospitals [11].

To avoid the harmful impact of sugars and to maintain their positive role, monitoring the concentration of sugars in food is needed, as well as choosing dietary sugars with low energy density that naturally occur in food—vegetables and fruits, including berries. According to this, lingonberries seem to be an appropriate source of dietary sugars [2,4]. Lingonberry (*Vaccinium vitis-idaea* L.) is a native plant to the boreal forest of North Eurasia and North America, nowadays generally accessible in most countries of Europe, especially in Scandinavia. Lingonberries can be consumed fresh, bought at the local markets, or cooked in the form of juices, jams, jellies, compotes, and syrups [12,13]. These berries are widely used in the human diet, especially sweetened products, which are more favorable for most consumers [14]. Fruits of this low-growing, evergreen shrub, belonging to the genus *Vaccinium* L., are popular not only because of their unique taste, but also because of their high level of healthy bioactive compounds. Lingonberries are considered to be a good source of flavonols, anthocyanins, phenolic acids, proanthocyanidins, free amino acids, vitamins, omega-3 fatty acids, and minerals [15,16]. A wide spectrum of biological activities of lingonberries has been determined. Lingonberries exhibit antimicrobial, anti-inflammatory, antioxidant, immunomodulatory, and antiproliferative activities and play a role in bacterial adhesion [12,17]. Products of lingonberries are increasingly marketed as a natural solution for the treatment of various conditions, particularly urinary tract infections [18].

However, the lingonberry’s composition and activity have not yet been fully investigated to date. Lingonberry is one of the least studied raw in the *Ericaceae* family; besides that, most studies were conducted in the Nordic countries and focused mainly on phenolic compounds [19]. Considering these berries’ popularity in food and health and wellness products, it is necessary to examine their sugar profile, due to potentially adverse effects. Lingonberries have historically been collected from the wild, and this is still mainly the case today. There are some cultivars produced, but there is no large-scale cultivation, and plant breeding of lingonberry is still in its infancy [20]. Cultivation of lingonberry can best meet the increased needs of plant material. To optimize horticulturally important traits, evaluation the phytochemical differences between cultivated and wild lingonberries is needed, as well as emphasis of factors such as optimal collecting time and environmental conditions leading to better yield.

To the best of our knowledge, there have been no comprehensive studies on sugar analysis of many lingonberry coenopopulations, considering phenological growth stages, altitude, and latitude of the berries’ collecting locations. The variations of identified sugars have been presented for the first time for the cultivars and lower taxa. This is the first report on sugar composition of *V. vitis-idaea* var. *leucocarpum*, which is a unique white berry-bearing variety that is included in the National Genetic Resources of Lithuania. The obtained results will be really important to breeders for developing new cultivars and, of course, as a part of the ongoing interest in nutritional and nutraceutical properties of food, the content of sugars in lingonberries will be of interest to dietitians and may be used in the pharmaceutical industry in developing new products for consumers with special dietary requirements. Our results can contribute to quality improvement of lingonberry products, leading to increased acceptability of consumers and market size. The findings on the content of fructose in lingonberries may disclose the necessity of further studies on fructose from lingonberries as a natural anti-adhesive agent. The sugar profile and individual sugar ratio can also serve as a fruit authenticity tool to prevent adulterations.

Therefore, our aim was to characterize the sugar composition in wild clones from Lithuania, in seven cultivars (’Erntedank’, ’Erntekrone’, ’Kostromička’, ’Kostromskaja rozovaja’, ’Rubin’, ’Sanna’, ’Sussi’) and lower taxa (*V. vitis-idaea* var. *leucocarpum*) of lingonberries, considering genetic and environmental factors.

## 2. Results

### 2.1. Method Validation

Linearity for all sugars was evaluated between 0.0625 and 4 mg/mL. Calibration equations with their linearity coefficients of detected sugars in lingonberries presented in Table 1. Limits of detection (LOD) of all tested sugars ranged between 11 and 18 µg/mL, and limits of quantification (LOQ) between 30 and 60 µg/mL. The precision values—repeatability and intermediate precision—were < 2% (relative standard deviation (RSD) of retention times < 1%, and RSD of peak areas < 2%). It could be concluded that our method gave acceptable precision for sugar measurements because the intra- and inter-day variations RSD for sugars were very low. The average recoveries of sugars were 98.08–102.15%, thereby confirming the accuracy of this analytical method. Resolution values were greater than 1.5 and selectivity values were greater than 1, ensuring that the sample components were well separated.

### 2.2. Qualitative Analysis of Sugars in Wild and Cultivated Lingonberries

**Figure 1:**
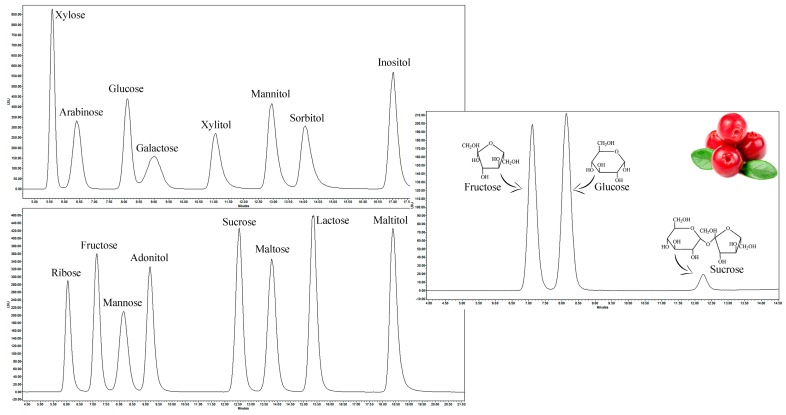
Chromatograms of reference compounds (on the **left**) and analyte (on the **right**).

Sixteen sugars, including mono- and disaccharides, as well as polyols, were searched in samples of wild and cultivated lingonberries. HPLC–ELSD results showed that only three sugars—fructose, glucose, and sucrose—were detected in all tested samples (Figure 1). Retention times of fructose, glucose, and sucrose were 7.109, 8.132, and 12.251 min, respectively.

### 2.3. Quantitative Analysis of Sugars in Wild Lingonberries

**Figure 2:**
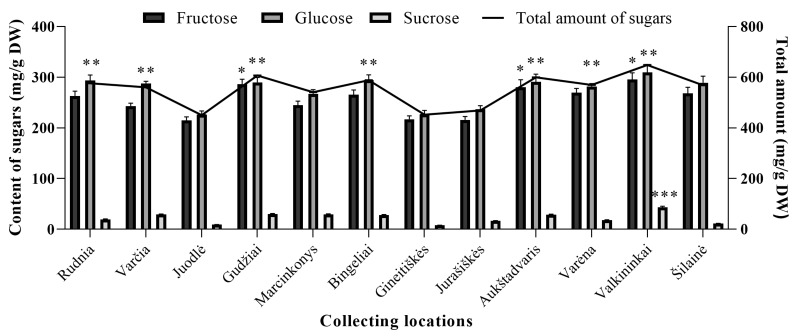
Composition of sugars in wild lingonberries clones. Bars marked with *, **, *** indicate the highest (*p* < 0.05) fructose, glucose, and sucrose amounts in lingonberries, respectively.

Fructose and glucose were the most abundant sugars in wild lingonberry extracts (Figure 2). These sugars contributed up to 98.01% of total sugars (the sum of identified sugar amounts). Contents of sucrose varied considerably among lingonberries from different collecting locations—the coefficient of variation (CV) was 42.61%—whereas the content of fructose and glucose varied only slightly (CV = 11.78% and 10.58%, respectively). The highest (*p* < 0.05) amounts of fructose were determined in Valkininkai (295.74 ± 13.80 mg/g dry weight (DW)), Gudžiai (286.63 ± 9.40 mg/g DW), and Aukštadvaris (280.58 ± 14.69 mg/g DW) forests, and the lowest (*p* < 0.05) in Juodlė (214.98 ± 6.92 mg/g DW), Jurašiškės (215.97 ± 6.42 mg/g DW), and Gineitiškės (217.10 ± 6.80 mg/g DW) forests. Contents of glucose were quite similar among different collecting locations, with the highest (*p* < 0.05) ones determined in lingonberries from Valkininkai forest (309.83 ± 15.18 mg/g DW), followed by Bingeliai, Aukštadvaris, Rudnia, and Marcinkonys forests. The lowest (*p* < 0.05) amounts of glucose accumulated lingonberries collected from Juodlė, Gineitiškės, and Jurašiškės forests (226.06 ± 7.28, 227.26 ± 7.12, and 236.77 ± 7.04 mg/g DW, respectively). Contents of sucrose ranged between 7.76 ± 0.24 mg/g DW in Gineitiškės forest (contribution 1.72% of total sugars) and 43.01 ± 2.51 mg/g DW in Valkininkai forest (contribution 6.63% of total sugars). Considering the sums of sugars, it was determined that lingonberries from Valkininkai forest accumulated the highest (*p* < 0.05) amount of total sugars (648.59 mg/g DW), meanwhile the lowest (*p* < 0.05) amount was determined in lingonberries from Juodlė, Gineitiškės, and Jurašiškės forests (450.23, 452.13, and 469.29 mg/g DW, respectively). Correlation analysis showed that sugar levels in wild lingonberries negatively correlated (*p* < 0.05) with latitudes and altitudes of their collecting locations.

**Figure 3:**
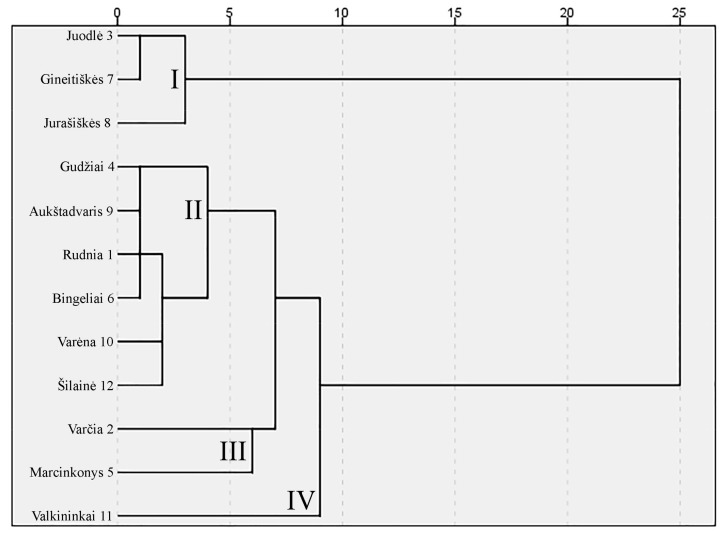
Dendrogram based on the amounts of sugars in wild lingonberry clones from different collecting locations.

The cluster analysis divided lingonberries from different collecting locations into four clusters, which differed statistically significant from each other (Figure 3). The first cluster consisted of the lingonberries from Juodlė, Gineitiškės, and Jurašiškės forests. Lingonberries collected from these forests distinguished themselves by the lowest (*p* < 0.05) amounts of all types of detected sugars. Cluster two was the largest and included lingonberries from Gudžiai, Aukštadvaris, Rudnia, Bingeliai, Varėna, and Šilainė forests. Wild clones from these forests could be characterized by higher than average amounts of fructose and glucose and low amounts of sucrose. Lingonberries from Varčia and Marcinkonys forests were attributed to the third cluster. These lingonberries, on the contrary to the second cluster, accumulated lower than average amounts of fructose and glucose and high amounts of sucrose. Lingonberries from the Valkininkai forest surpassed lingonberries from other collecting locations by the highest (*p* < 0.05) contents of all sugars, and were attributed to the fourth cluster. These results highlight differences between wild clones of lingonberries and their adaptability to growing and environmental conditions in different locations.

### 2.4. Quantitative Analysis of Sugars in Cultivated Lingonberries

**Figure 4:**
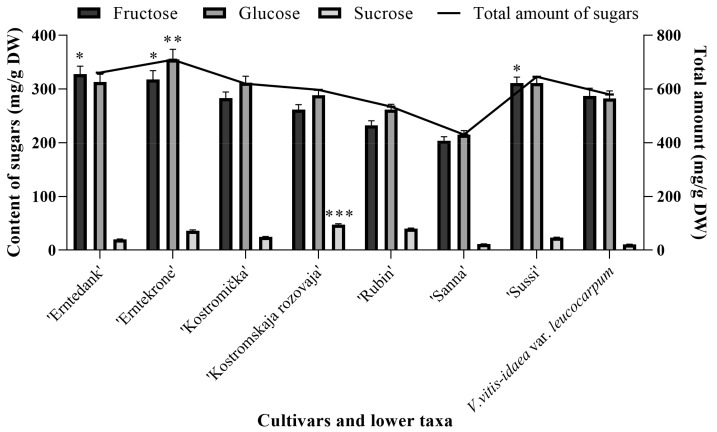
Composition of sugars in cultivated lingonberries. Bars marked with *, **, *** indicate the highest (*p* < 0.05) fructose, glucose, and sucrose amounts in lingonberries, respectively.

Similarly to wild clones, the predominant sugars in cultivars and lower taxa of lingonberries were fructose and glucose (Figure 4). Fructose contributed 43.51–49.49% and glucose contributed 47.40–50.29% of total sugars in different cultivars and lower taxa of lingonberries. The highest contribution of glucose and fructose was found in *V. vitis-idaea* var. *leucocarpum* (98.16%), and the lowest one in ‘Kostromskaja rozovaja’ (92.08%). CV of fructose and glucose among tested cultivars and lower taxa were 15.55% and 14.27%, respectively, indicating that the contents of these sugars between cultivated lingonberries did not differ extremely. Significantly the highest contents of fructose were determined in ‘Erntedank’ (327.64 ± 15.01 mg/g DW), ‘Erntekrone’ (317.72 ± 16.57 mg/g DW), and ‘Sussi’ (311.01 ± 11.17 mg/g DW); meanwhile, the lowest (*p* < 0.05) were in ‘Sanna’ (203.82 ± 7.32 mg/g DW) and ‘Rubin’ (232.47 ± 8.35 mg/g DW) cultivars of lingonberries. The content of glucose among tested cultivars and lower taxa of lingonberries ranged between 214.78 ± 7.72 mg/g DW (in ‘Sanna’ cultivar) and 355.24 ± 18.53 mg/g DW (in ‘Erntekrone’ cultivar). Amounts of sucrose (1.84–7.92% of total sugars in different cultivars and lower taxa of lingonberries) were considerably lower than that of other sugars, but varied within a wide range (CV = 49.85%). Significantly, the highest content of sucrose was found in ‘Kostromskaja rozovaja’ cultivar (47.29 ± 1.71 mg/g DW), whereas the lowest (*p* < 0.05) in ‘Sanna’ cultivar (11.46 ± 0.41 mg/g DW) and *V. vitis-idaea* var. *leucocarpum* (10.68 ± 0.54 mg/g DW). It was noticed that ‘Erntekrone’ cultivar accumulated the highest (*p* < 0.05) amount of total sugars (708.78 mg/g DW), whilst a lower amount of even more than one and a half times was determined in ‘Sanna’ cultivar.

**Figure 5:**
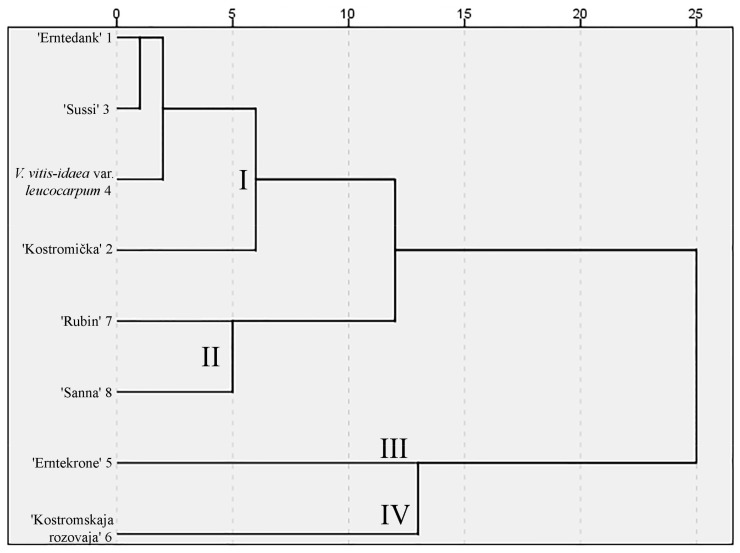
Dendrogram based on the amounts of sugars in cultivated lingonberries.

After hierarchical cluster analysis, the lingonberry cultivars and lower taxa fruit samples were grouped into four significantly different clusters (Figure 5). The first cluster was distinguished by the highest amounts of total sugars, but the lowest of sucrose. The lingonberry cultivars attributed to this cluster were German cultivar ‘Erntedank’, Swedish cultivar ‘Sussi’, and Russian cultivar ‘Kostromička’. Lingonberries of Russian (‘Rubin’) and Swedish origin (‘Sanna’) accumulated the lowest amounts of fructose and glucose and were attributed to the second cluster, whereas the highest amounts of these two sugars were determined in a cultivar of German origin (‘Ertekrone’), which was attributed to the third cluster. The Russian origin ‘Kostromskaja rozovaja’ was attributed to the fourth cluster, which was characterized by the highest (*p* < 0.05) amount of sucrose and the lowest amount of total sugars. The results of the cluster analysis of cultivated lingonberries indicate genetic variations in the levels of sugars.

### 2.5. Comparison of Sugars Between Wild and Cultivated Lingonberries

**Figure 6:**
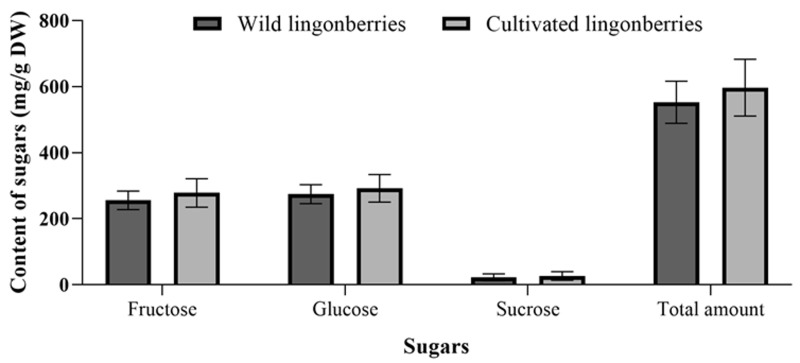
Average amounts of sugars in wild and cultivated lingonberries.

The same sugars with dominant fructose and glucose were found either in wild or cultivated lingonberries, collected at the same time—berry formation stage in 2017 (Figure 6). The average amounts of total sugars were 552.58 ± 63.57 mg/g DW in wild and 596.92 ± 85.78 mg/g DW in cultivated lingonberries. Compared with wild lingonberries, cultivated ones accumulated 1.1, 1.1, and 1.2 times higher amounts of glucose, fructose, and sucrose, respectively. Nevertheless, an independent samples *t*-test showed that there were no statistically significant differences between sugar amounts in the wild and cultivated lingonberries groups. Hence, domestication of lingonberries, genetical changes resulted from human selection, and also presently used constant fertilization, irrigation, and other monitored cultivation conditions had only a slight effect on the accumulation of sugars.

### 2.6. Sugars of Lingonberries During the Growing Season

**Figure 7:**
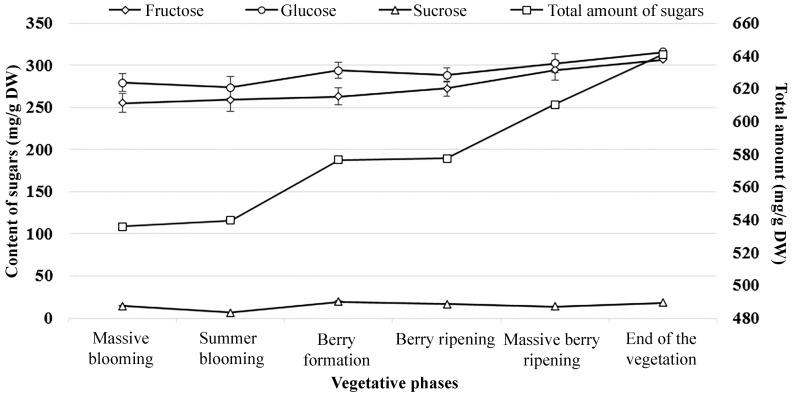
Content of sugars during different vegetative phases of lingonberries.

The results show that levels of sugars varied unevenly during the growing season (Figure 7). The highest (*p* < 0.05) amount of total sugars was found in lingonberries collected at the end of the vegetation (640.83 mg/g DW). Fructose, glucose, and sucrose levels in berries since the massive blooming stage till the end of the vegetation increased 20.23%, 18.56%, and 26.18%, respectively. Interestingly, the content of sucrose was the highest at the berry formation stage, but this content did not differ statistically significantly from the content accumulated at the end of the vegetation, and had no significant contribution to the total amount of sugars. According to the results, it is apparent that the amounts of sugars are increasing during the growing season, and lingonberries collected at the end of the vegetation are sweeter than those of the beginning of the vegetation.

## 3. Discussion

Lingonberries are described as very sour and quite tart berries with a little bit of sweetness. The flavor is very similar to cranberries. Organoleptic properties of lingonberries, like other fruits, are mainly determined by volatile compounds, sugars, organic acids, and their ratio [21]. Since sugars in lingonberries affect consumer acceptability, a number of studies have been accomplished.

Mikulic-Petkovsek et al. analyzed various species of berries by the HPLC–RI method of analysis and detected glucose (37.9 ± 1.32 mg/g fresh weight (FW)), fructose (29.2 ± 0.71 mg/g FW), and sucrose (4.10 ± 0.45 mg/g FW) in wild lingonberries. Compared with other tested berries, the sugar level in lingonberries was just moderate. Similar contents of sugars were found in wild blackberries, red gooseberries, black mulberries, goji berries, and wild-grown elderberries [8]. Almost the same amounts of sugars—up to 29, 36, and 2 mg/g FW of glucose, fructose, and sucrose, respectively—were determined earlier in lingonberries, bought from the local retail shop [22]. The sugars were also found in the lingonberry juices, extracted with a hydraulic press. Viljakainen et al., by using the HPLC–RI method of analysis, found that amounts of fructose and glucose in Finnish lingonberry juices were almost equal (42.30 ± 0.27 and 42.38 ± 0.39 mg/mL, respectively) and contributed up to 98.6% of total sugars, whereas amounts of sucrose were very low (1.17 ± 0.01 mg/mL). Assessed amounts of total sugars were slightly higher in lingonberry juices than in juices of bilberry, cloudberry, blackcurrant, and strawberry, and almost two times higher than those of redcurrant, cranberry, and black crowberry [23]. Recent studies have shown that sugars in lingonberry juices could be detected by a sensitive spectrophotometric method using enzymatic assay kits specific for these carbohydrates. It was proclaimed that squeezed lingonberry juices had 38.9 ± 0.43 mg/mL of fructose and 45.4 ± 0.71 mg/mL of glucose. Contents of sugars were similar to those of elderberry juices, but higher (*p* < 0.05) than in cornelian cherry juices [24]. One more report revealed that bioprocessing unaffected, fully riped Finnish lingonberries accumulated high amounts of fructose (260 ± 0.01 mg/g DW), glucose (248 ± 0.03 mg/g DW), and sucrose (23.0 ± 0.01 mg/g DW) [21].

Our determined sugar amounts might seem higher than in previous studies, except the latter one, in which observed sugar composition was consistent with the results of the present study. It can be explained that in all of the researches mentioned above, except the latter one, the results were expressed for fresh raw material, meanwhile in ours for dry raw material (lyophilized lingonberries). The differences in contents of sugars also can be attributed primarily to the morphotype of lingonberries, as well as geographical locations, prevailing climatic conditions, fruit ripeness, their collecting date, and diversity of processing, extraction, and sugar analysis.

Notwithstanding the distinctions between sugar concentrations, contributions of fructose, glucose, and sucrose of total sugars were partly consistent in previous studies, and a number of reports proved that the main sugars of lingonberries are fructose and glucose. The amounts of other sugar components could appear only after hydrolysis of polysaccharide fraction. Ross et al., by using gas–liquid chromatography with FID detector, found impressively high amounts of arabinose, xylose, and galactose, and lower amounts of mannose, fucose, and rhamnose in water-extractable polysaccharides fraction from Northern Manitoba lingonberries [25]. Some of these sugars were looked at in the present study as well, but results showed that they cannot be found in lingonberries as free sugars.

Results of the present study and literature data revealed that sugar concentration in lingonberries is higher than in the most popular berries, like bilberries, strawberries, and cranberries. However, the sweet taste is hidden because of the high organic acid content. Several studies have shown that lingonberries accumulate high amounts of citric, fumaric, and shikimic, and lower amounts of tartaric, benzoic and malic acids, which result in pH decreasing of lingonberries [8,21,23]. Bioprocessing of lingonberries with enzymes, lactic acid bacterias, yeast, or their combination has an important impact on sugars composition, and enzyme treatment could be a potential tool for decreasing the acidic flavor of lingonberries [21].

It is acknowledged that lingonberries are indigenous to the sandy, northern, temperate, boreal forests; they prefer light and well-drained, porous, acidic (pH range between 4.3 and 5.5) soils. However, lingonberries are not demanding—they are resistant to temperature fluctuations, require very little water, and can grow in very different habitats within its extensive natural range, from dry oligotrophic pinewoods to raised bogs [26,27,28]. Lithuanian boreal forests seem to be a suitable place for the growth of lingonberries. Considering the sugar amounts in lingonberries from different collecting locations of Lithuania, it was noticed that the amounts of sugars in medium humidity, very infertile, pine tree with full sun or partial shade forests (e.g., Varėna, Valkininkai, Aukštadvaris, Marcinkonys) were higher (*p* < 0.05) than in medium humidity, medium fertility, with large variety of tree species shaded forests (e.g., Gineitiškės, Jurašiškės). Thus, our results are in accordance with the literature data and indicate that lingonberries accumulate higher amounts of compounds in sunny, dry tree sites, and even very infertile land can be a great growth place for lingonberries. Detailed studies are needed on the accumulation of other bioactive compounds in different soils, determining the organic and mineral composition of the soil.

Our determined amounts of sugars in different cultivars of lingonberries were partly consistent with their description of the flavors and yield. Cultivars in which berries are characterized as weakly acidic or sweet-and-sour in taste were distinguished by the highest amounts of sugars (‘Erntedank’, ‘Sussi’, ‘Kostromička’, which were attributed to the same cluster, and ‘Ertekrone’, which was attributed to a separate cluster). Meanwhile, the cultivars in which berries are described as having a sour taste and producing poor fall crop accumulated the lowest amounts of fructose and glucose (‘Sanna’ and ‘Rubin’, which were attributed to the same cluster) [26,29].

The composition of bioactive compounds in cultivated lingonberries is the subject of numerous studies. Lee et al. analyzed five different cultivars, including Swedish cultivars ‘Sanna’ and ‘Sussi’, which were also examined in our study. They assessed that lingonberries of these cultivars accumulated the lowest (*p* < 0.05) amounts of amino acids and moderate amounts of anthocyanins, total phenolics, and total tannins [13]. The lowest sugar productivity by ‘Sanna’ and one of the highest by ‘Sussi’ was observed in the present material. Phenolic compounds from lingonberry leaves within the same cultivars and lower taxa as in the present study were investigated previously by us. The greatest amounts of phenolics were found in the leaf extracts from ‘Rubin’ and ‘Kostromskaja rozovaja’ cultivars, whereas the lowest ones in ‘Erntedank’, ‘Erntesegen’, and ‘Sanna’ cultivars. The cluster analysis revealed that, according to the composition of phenolic compounds, the clusters were related to the countries of origin, especially with German and Russian origin cultivars [30]. According to the present findings, ‘Rubin’ and ‘Kostromskaja rozovaja’ cultivars accumulated just moderate amounts of sugars in berries, ‘Sanna’ with the lowest (*p* < 0.05), and ‘Erntedank’, ‘Erntesegen’ with the highest ones. Furthermore, there was no link between the cultivar country of origin and the quantities of sugars in berries. Contents of sugars do not seem to correlate with the contents of other compounds in the same cultivars and lower taxa of lingonberries. The productivity of cultivars in terms of sugars and in terms of other compounds is probably different. However, it is hard to compare the results from the earlier and present study, because of the different studied cultivars or raw materials.

Taking into account the importance of genetic differences and control of cultivation conditions, it is anticipated that amounts of bioactive compounds should be different among wild clones and cultivated plants. Notwithstanding, we found that there were no statistically significant differences in sugar amounts between cultivated and wild lingonberries. Several researchers found higher levels of phenolic compounds in wild fruits, meanwhile, the concentration of total sugars was quite similar between cultivated and wild fruits, as in our study [8,31,32]. This information could be relevant for breeders that are interested in sugar levels in the development of new cultivars, and it indicates that they should look at some other cultivation techniques that may help affect the sugar content. The successful development of lingonberry cultivars would increase the market size.

Altitude and latitude of location have an impact on temperature and solar radiation. As the latitude of a location increases, it receives less sunlight, whereas increasing altitude results in a decrease in pressure and thus in temperature. It is anticipated that most plants may adapt to higher latitudes and altitudes. Harsh weather conditions could affect processes associated with plant development and significantly enhance the biosynthesis of bioactive compounds [33,34]. Vyas et al. determined that amounts of secondary metabolites—anthocyanins, proanthocyanidins, and total antioxidant activity—of wild lingonberries positively correlated with latitude and altitude of the berries’ collecting locations [35]. Our study showed contrary results—higher altitude and latitude, less sunlight and lower temperature reduced sugar production in lingonberries. Consequently, we suggest that the synthesis of primary metabolites does not intensify under the harsh weather conditions.

The variability and contents of bioactive compounds in plants depend on many factors, such as the already discussed genetic and environmental factors, cultivation conditions, processing, extraction method, and also maturity stage [36]. Numerous studies have been conducted to determine the amounts of bioactive compounds in various berries during the growing season, thus finding out the optimal collecting time [36,37,38]. Hence, we figured out that the changes in the sugar levels during the growing season were apparent in lingonberries and the highest amounts accumulated at the end of the vegetation. So, late September would be the optimal collecting time for those who prefer sweeter berries. Analysis of the relationship of sugar amounts in lingonberries and consumer expectations would help to develop the best quality standards for collecting dates.

Although there are considerable amounts of sugars in lingonberries, there is no need to worry about high sugar intake with these berries leading to deleterious health effects. These effects may occur if only more than about 1.25 kg of fresh lingonberries would be eaten daily, and more than 2.5 kg/day of fresh lingonberries would contribute to weight gain [6]. Meanwhile, the moderate intake of lingonberries may trigger satiety and promote a positive energy balance due to sugars; also lingonberry inclusion in the diet predisposes prevention of various human chronic diseases, because of the richness of the phenolic antioxidants [39]. Published papers show the potential benefit of lingonberries against diabetes and hypertension. These berries can inhibit α-amylase, α-glucosidase, anti-diabetic agent acarbose, and significantly enhance glucose uptake in human liver cells, decreasing glycemia and insulin levels [40,41]. Kivimäki et al. reported that lingonberry juices at small concentrations affect plasma inflammatory markers, clinical chemistry variables, and may lower blood pressure in long-term treatment [42]. Furthermore, lingonberry extracts with strong antioxidant function consumed orally or topically can protect dermal collagen protein, reduce the production and activity of elastinase, relieve skin wrinkles and colored spots, and thus improve skin conditions [43,44]. Therefore, products of lingonberries are promising beauty and health therapeutic candidates in the cosmetic and pharmaceutical industries.

## 4. Materials and Methods

### 4.1. Chemicals and Solvents

Analytical and chromatographic grade reference compounds were used for this study: Xylose, arabinose, glucose, galactose, xylitol, mannitol, sorbitol, inositol, ribose, fructose, mannose, adonitol, sucrose, maltose, lactose, and maltitol were purchased from Sigma-Aldrich GmbH (Steinheim, Germany). HPLC grade acetonitrile was obtained from Sigma-Aldrich GmbH (Steinheim, Germany), and purified deionized water (18.2 mW/cm) was produced using the Millipore (Millipore, Bedford, MA, USA) water purification system.

### 4.2. Plant Material

#### 4.2.1. Wild Lingonberries

The description of collecting locations of tested lingonberry wild clones is displayed in Table 2. These collecting locations of wild clones differed from each other by soil moisture and degree of yield, therefore by the quality of growing conditions. The soil of lingonberry collecting locations was medium humidity, very infertile, with predominant Scots pine (*Pinus sylvestris* L.) tree species in Varėna, Valkininkai, Aukštadvaris, Marcinkonys, and Juodlė forests; medium humidity, infertile, with predominant Silver birch (*Betula pendula* Roth) and European spruce (*Picea abies* (L.) H. Karst.) tree species in Rudnia and Gudžiai forests; and medium humidity, medium fertility, with a large variety of tree species in Gineitiškės, Varčia, and Jurašiškės forests.

Samples of wild lingonberries were collected in berry formation stage (third decade of August, 2017) in the twelve forests mentioned above, and also during different vegetative phases: Massive blooming (2 August, 2017), summer blooming (12 August, 2017), berry formation (21 August, 2017), berry ripening (2 September, 2017), massive berry ripening (16 September, 2017), and at the end of the vegetation (26 September, 2017) in Rudnia forest. These collecting times were chosen according to the lingonberry vegetative phases in Lithuania [26].

Lithuania, a flat country overlooking the Baltic Sea, has a humid continental climate, which can be described as a typical European continental influenced climate with warm, dry summers and fairly severe winters. Agricultural land covers more than 50% of Lithuania; forested land consists of about 28%, with 1.8 million ha. Lithuania is situated within the so-called mixed forest belt, with a high percentage of broadleaves and mixed conifer–broadleaved stands. The average solar radiation during the lingonberries’ growing season from April to September of 2017 was 490 MJ/m^2^ and ranged between 259 MJ/m^2^ in September and 710 MJ/m^2^ in May; meanwhile, the average precipitation was 61 mm and ranged between 20 mm in May and 125 mm in September. Average temperatures varied from 5 °C in April to 17 °C in August. The lowest moisture (53%) during the lingonberries’ growing season in 2017 was determined in May, meanwhile the highest ones—75% and 80%—in August and September, respectively, when berries were collected for this study. It should be noted that the duration of sunshine in 2017 in Lithuania was about 1600 h, and it is much lower compared with previous years; meanwhile, the average temperature of 2017 years was 7.6 °C, which is 0.7 °C above standard climate rate. The meteorological data were obtained from the archive of the Lithuanian Hydrometeorological Service under the Ministry of Environment.

#### 4.2.2. Cultivated Lingonberries

The cultivated lingonberries were collected in the field collection of the Botanical Garden of Šiauliai University (55°55′57′′N, 23°16′59′′E (WGS)). The following seven cultivars of lingonberry were tested: Russian cultivars—’Kostromskaja rozovaja’ (registered in 1995), ‘Kostromička’ (1995), and ‘Rubin’ (1995); Swedish cultivars—’Sanna’ (1987) and ‘Sussi’ (1986); and German cultivars—’Erntedank’ (1975) and ‘Erntekrone’ (1978). Also, one variety (taxonomic rank between subspecies and form) of lingonberries—*V. vitis-idaea* var. *leucocarpum* Asch. et Magnus —was included in the study. Lingonberries belonging to this variety distinguish themselves by white berries, and the first time they were found was in 1993 in the forest of Svencioneliai district, Lithuania [27,45].

Lingonberries were cultivated in a partially shaded place, with acidic and well-drained soil. According to meteorological situation, fertilization and irrigation were periodically applied. The dynamics of meteorological factors during the growing season of cultivated lingonberries corresponded to those of wild clones. Fruits of different cultivars and lower taxa of lingonberries were collected at the end of August 2017 (berry formation stage).

### 4.3. Sample Preparation and Extraction of Sugars

After collecting, lingonberries were immediately frozen and subjected to lyophilization in ZIRBUS sublimator 3 × 4 × 5/20 (ZIRBUS Technology, Bad Grund, Germany) at a pressure of 0.01 mbar (condenser temperature, –85 °C). To grind lyophilized berries to a fine powder, the Retsch 200 mill (Haan, Germany) was used. Lyophilizates of lingonberries were comprised of 0.5 kg berries of each cultivar, lower taxa or wild clones from different forests. All obtained results were re-calculated for dry raw plant material.

One gram of the ground lyophilized lingonberries was added to 15 mL of distilled water in the conical flask and extracted three times in an ultrasonic bath (Elmasonic P, Singen, Germany) for 10 min. After the extraction, the homogenates were centrifugated for 5 min at 8500 rpm in a Biofuge Stratos centrifuge, and obtained supernatants were filtered through a membrane filter with a pore size of 0.22 μm (Carl Roth GmbH, Karlsruhe, Germany).

### 4.4. Qualitative and Quantitative Analysis of Sugars by HPLC–ELSD Method

The determination of sugar contents was performed using the Waters 2695 Alliance system (Waters, Milford, MA, USA) equipped with a Waters 2424 evaporative light-scattering detector (ELSD). Separation of sugars was carried out using Shodex SUGAR SZ5532 (Showa Denko KK) column (150 × 6.0 mm), according to the methodology described by Zymone et al. [46]. The gradient consisted of eluent A (water) and B (acetonitrile) and followed: 0–5 min—81% B, 5–20 min—81–70% B, 20–22 min—70% B, 23 min—81% B, with the eluent flow rate—1 mL/min and injection volume—10 µL. As the ELSD nebulizer gas (25 psi), nitrogen was used and tube temperature was set to 60 °C. Chromatographic peak identification was carried out by comparing the retention times of the analyte and reference compounds. For sugar quantification, calibration curves were constructed.

### 4.5. Method Validation

The analytical HPLC–ELSD method was validated in terms of linearity, LOD, LOQ, precision accuracy, and system suitability according to the ICH Q2(R1) guidelines [47]. To find out the linearity, standard solutions of authentic samples of sugars were prepared. The standard curves were based on five concentrations, each analyzed in triplicate. LOD and LOQ were determined based on a signal-to-noise ratio (S/N). The detection limit was defined as the concentration that gave a signal-to-noise ratio (S/N) >3 and the quantification limit as the concentration that gave S/N >10. The precision of the method was evaluated by measurement in intra-day (for repeatability) and inter-day (for intermediate precision) variability tests, calculating the relative standard deviation (RSD) of peak areas or retention times. Accuracy of the method was expressed as percent recoveries, which were studied by adding known amounts of standards to the samples. System suitability parameters—resolution and selectivity—were calculated using Empower™ System Suitability software.

### 4.6. Statistical Analysis

Statistical analysis was conducted using SPSS 21.0 (SPSS Inc., Chicago, IL, USA) and Microsoft Office Excel 2010 (Microsoft, Redmond, WA, USA). The amounts of sugars were expressed as the mean (M) of three measurements ± standard deviation (SD). Analysis of variance (ANOVA) with Tukey’s HSD post-hoc test and independent samples t-test were performed to determine the significant differences among the wild and cultivated lingonberries (*α* = 0.05). Correlations were tested using Pearson’s correlation test. Hierarchical cluster analysis was carried out using the centroid clustering method with squared Euclidean distances.

## 5. Conclusions

Observed sugar amounts in lingonberries are not dangerously high, and consequently, do not seem to be hazardous for consumers of food or health and wellness products of lingonberries. The same sugars with dominant fructose, glucose, and low amounts of sucrose were found in wild and cultivated lingonberries. As a lingonberry product authentication tool, these simple sugars could be searched to prevent adulterations. The presence of moderate levels of fructose in lingonberries reveals potential type 1 fimbriae inhibiting activity and relevance for further studies. Intraspecific variability was detected when comparing the content of sugars in wild clones or cultivated lingonberries, regarding to phenological growth stages. The highest contents of sugars were observed in berries collected at the end of the vegetation—full ripening stage, from sunny, well-drained soil locations with higher solar radiation and temperature. Consideration of these factors may result in lingonberry yields with preferred sugar levels. Naturally sweeter food products of lingonberries would reduce the need for added sugars, whereas reduced sugar levels in orally consumed pharmaceuticals or nutraceuticals would increase the acceptability of consumers.

## Figures and Tables

**Table 1:** The linearity of calibration curves of lingonberry sugars.

Component	Calibration Equation	Coefficient of Determination *R*^2^	Coefficient of Correlation *R*
Fructose	Y = 1.80 X + 44.9	0.9998	0.9999
Glucose	Y = 2.00 X − 26.3	0.9876	0.9938
Sucrose	Y = 1.77 X + 82.2	0.9999	0.9999

**Table 2:** Collecting locations of wild lingonberries, with their latitudes, longitudes, and altitudes.

Forest	Latitude (°)	Longitude (°)	Altitude (m)
Rudnia	54.40	24.49	137
Gineitiškės	54.49	24.39	155
Marcinkonys	54.07	24.43	123
Varėna	54.29	24.44	136
Valkininkai	54.36	24.85	116
Gudžiai	54.36	24.43	136
Bingeliai	54.15	24.25	112
Šilainė	54.08	23.71	135
Varčia	54.32	24.21	148
Aukštadvaris	54.57	24.61	167
Juodlė	55.83	22.94	141
Jurašiškės	54.10	23.89	135
